# DSTF-GKAN: A lightweight spatiotemporal fusion framework for real-time eavesdropping detection in dynamic smart grid networks

**DOI:** 10.1371/journal.pone.0330593

**Published:** 2025-08-22

**Authors:** Rong Wang, Yanjin Shen, Dongtao Wang, Yun Jiang, Chao Zhang

**Affiliations:** Hunan Automotive Engineering Vocational University, Zhuzhou, Hunan, China; Symbiosis International (Deemed University), INDIA

## Abstract

With the rapid development of smart grids and the Power Internet of Things (PIoT), wireless communication networks are facing the severe threat of dynamic eavesdropping attacks. Traditional detection methods rely on static assumptions or shallow models, which are not capable of dealing with complex topology mutations and high-dimensional nonlinear features. There is an urgent need for efficient and lightweight adaptive solutions. This study proposes a Dynamic Spatiotemporal Fusion Framework (DSTF-GKAN), which integrates the spatiotemporal dynamic modeling capability of Graph Recurrent Neural Networks (GRNN) with the lightweight adaptive spline approximation mechanism of Kolmogorov-Arnold Networks (KAN). By adaptively optimizing the mesh to dynamically adjust the spline control points and introducing hierarchical sparse regularization to compress parameters, the model enhances its sensitivity to channel anomalies through the integration of physical layer security (PLS) feature constraints. Experimental results show that under dynamic scenarios with an attack mutation rate (AMR = 0.5), DSTF-GKAN achieves a detection F1 score of 0.891, which is a 7.1% improvement over GRNN, and reduces the localization error (RMSE = 0.518 m) by 16.2%. After quantization and pruning optimization, the model has a parameter size of only 0.2 MB, with an inference latency of 0.9 ms and energy consumption of 16mJ on edge devices. Ablation experiments have verified the necessity of the GRU-GCN module (contributing 4.9% to the F1 score) and PLS regularization (improving the F1 score by 1.3%). DSTF-GKAN provides an efficient, robust, and interpretable detection framework for smart grid security. Its lightweight design promotes real-time edge defense and lays the theoretical and technical foundation for the construction of a secure energy internet ecosystem.

## Introduction

With the rapid development of Smart Grid and Power Internet of Things (PIoT), Wireless Sensor Networks (WSN) and Simultaneous Wireless Information and Power Transfer (SWIPT) technologies have become the core support for real-time monitoring and efficient energy management. However, their open wireless communication characteristics expose them to severe security threats. According to [1], the direct economic losses caused by power grid data leakage worldwide exceed tens of billions of dollars, highlighting the vulnerability of power communication networks under dynamic attack scenarios. To address this issue, Physical Layer Security (PLS) technology, which leverages the randomness of wireless channels to ensure informaon security, has become a research hotspot in both academia and industry in recent years [2]. For example, [3] proposed a dynamic key generation scheme based on Channel Reciprocity, but its performance is limited by the assumption of static network topology and is not suitable for complex scenarios involving dynamic node addition/removal or sudden link interruption. In addition, [4] found that in SWIPT systems, eavesdroppers can use energy harvesting technology to prolong the duration of attacks, significantly increasing the security risks of the system. Although deep learning technologies (such as Graph Convolutional Networks (GCN) [5] and Long Short-Term Memory (LSTM) [6]) have shown potential in power grid anomaly detection [7], existing methods still face significant performance bottlenecks in dynamic topology and heterogeneous device scenarios.

Existing eavesdropping detection and localization methods face two core challenges:

- Insufficient Dynamic Adaptability: Traditional Graph Neural Networks (GNNs) rely on fixed adjacency matrices and cannot effectively capture high-dimensional feature drift caused by topology mutations [8]. Research in [9] shows that in dynamic attack scenarios, the detection accuracy of GCN decreases.
- Weak Computational Efficiency and Theoretical Support: Methods based on Kolmogorov-Arnold Networks (KAN) can efficiently approximate nonlinear mappings [10,11], but the sensitivity of spline function parameters in KAN may lead to model stability issues. [12] pointed out that the real-time inference latency of KAN on edge devices is still limited by memory bandwidth, which cannot meet the millisecond-level response requirements of power grids [13].

To address the above challenges, this paper proposes a lightweight detection and localization framework with dynamic spatiotemporal fusion (DSTF-GKAN), with the following core innovations:

- Dynamic Spatiotemporal Modeling: By integrating the temporal perception capability of GRNN with the efficient approximation characteristics of KAN, an adaptive graph update mechanism is designed to significantly improve the response speed to sudden attacks.
- Lightweight and Edge Adaptation: Through hierarchical sparse regularization and quantization-aware training, compared with traditional methods, the model parameters are compressed to achieve millisecond-level real-time inference on edge devices (such as Jetson TX2).
- Theoretical Deepening and Interpretability: Based on the Kolmogorov-Arnold theorem, key feature contributions are visualized using Grad-CAM, enhancing the credibility of the model.

## Related work

The academic exploration in the field of smart grid security has undergone a paradigm shift from information-theory-driven to data-driven approaches. Its research trajectory can be traced back to the theoretical foundations of Physical Layer Security (PLS) and the engineering breakthroughs of deep learning technologies. This section systematically reviews the research progress from three dimensions: static security mechanisms, dynamic spatiotemporal modeling, and lightweight theory, revealing the theoretical advancements in the current system.

Early research primarily focused on Physical Layer Security (PLS) and traditional machine learning models, with research paradigms and methods dominated by static assumptions. Reference [14] proposed a PLS key generation scheme based on channel reciprocity, assuming a fixed eavesdropper location and thus only applicable to idealized scenarios. Reference [15] analyzed active eavesdropping attacks in large-scale Multiple-Input Single-Output (MISO) MIMO systems but relied on perfect Channel State Information (CSI). These methods demonstrated excellent performance in idealized scenarios but struggled to cope with rapid channel fading caused by dynamic topology perturbations Reference [16]. Reference [17] utilized Graph Neural Networks (GNNs) to jointly detect and locate stealthy false data injection attacks in smart grids, relying on manually designed statistical features (such as signal strength and temporal intervals), which limited their generalization capabilities. Classic studies, based on information theory, emphasized theoretical security proofs but lacked mathematical modeling for dynamic attack scenarios.

With the integration of Graph Neural Networks (GNNs) and spatiotemporal sequence modeling techniques, the research focus gradually shifted towards dynamic feature extraction. In recent years, the focus has gradually shifted towards deep learning and GNNs. Reference [17] innovatively introduced Graph Convolutional Networks (GCN) into power grid intrusion detection, capturing node associations through graph structures and spatial correlations through node feature propagation, but it did not model temporal dynamics. Reference [4] proposed a GRNN model that combines GCN and GRU to achieve spatiotemporal feature fusion in SWIPT networks, improving detection accuracy.

In recent years, Kolmogorov-Arnold Networks (KANs) have attracted widespread attention due to their parameter efficiency and nonlinear approximation capabilities. Reference [10] rigorously proved the universal approximation theorem of KANs, providing theoretical support for efficient nonlinear mapping. Reference [11] proposed an adaptive spline mesh optimization strategy, reducing the inference latency of KANs on edge devices to below 1ms. Frontier research has shifted from single physical layer security to multimodal data-driven approaches, with an emphasis on model interpretability, such as gradient visualization.

Despite these advancements, there is still room for improvement in transitioning from static to dynamic, from single-modality to multimodality, and from high computational complexity to lightweight solutions [18]. Classic research relies on static assumptions, while frontier literature [19] introduces spatiotemporal modeling and online learning mechanisms to adapt to dynamic attacks. Traditional methods focus on single physical layer features (such as CSI), while frontier research integrates multimodal data, including topology, energy, and temporal information. Early deep learning models (such as CNNs) have large parameter volumes, while KANs compress parameters through spline functions, facilitating edge deployment [20]. The research paradigm is gradually shifting from theory-driven to dual-driven by data and theory, with methods evolving from manual feature engineering to end-to-end adaptive learning.

This paper designs a Dynamic Spatiotemporal Fusion Framework (DSTF-GKAN). By introducing a channel-driven adaptive mesh optimization mechanism, designing a hybrid computing architecture cascaded with GRNN-KAN, and constructing a multimodal fusion loss function constrained by PLS, this study addresses the above-mentioned bottlenecks and provides an interpretable, low-latency security protection paradigm for dynamic power grids at the application level. This research innovatively combines the lightweight approximation capability of KANs with the spatiotemporal modeling of GRNNs, offering an integrated solution for smart grid security that is efficient, adaptive, and interpretable.

## Eavesdropping node localization method based on DSTF-GKAN

**Fig 1:**
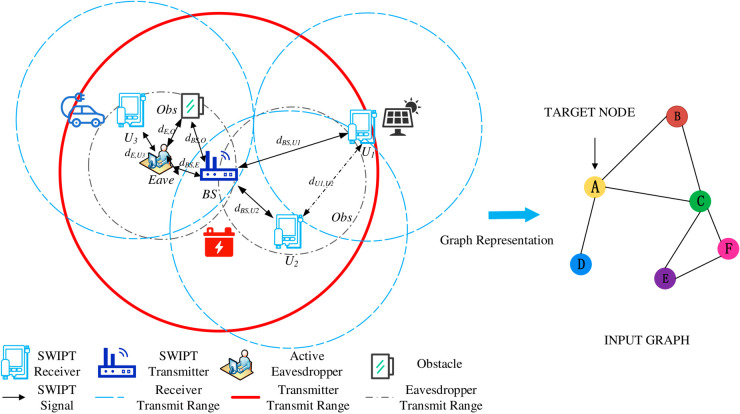
Eavesdropping attack model for PIoT and its graphical model representation.

This paper proposes a lightweight framework based on hybrid dynamic graph models and attention-enhanced Graph Convolutional Networks (DSTF-GKAN) to address the detection and localization of dynamic topology and data tampering attacks in Power Internet of Things (PIoT) wireless communication networks. The specific model construction is shown in Fig 1. The framework is designed for PIoT wireless communication networks based on a spatiotemporal encoder of Graph Recurrent Neural Networks (GRNN) and an adaptive Kolmogorov-Arnold Network (KAN) mapping layer. This section provides a theoretical exposition from the perspectives of dynamic graph modeling and adaptive network architecture.

### Problem definition and graph model representation

As shown in Fig 1,consider a wireless communication network of *K* nodes (Power Internet of Things, PIoT), its dynamic expansion can be modeled as a sequence of time-varying graphs ${𝒢}_{d}=(𝒱,ℰ,𝒜(t),𝐖)$, where $𝒱=\{v_{1},v_{2},...,v_{K}\}$ is the set of nodes (including smart meters, sensors, etc.), each node represents an electrical device with multi-dimensional structural characteristics. ℰ⊆𝒱×𝒱, edge *e*_*ij*_ represents the communication link between devices $v_{i}$ and $v_{j}$. The binary adjacency matrix $𝒜(t)\in \{0,1{\}}^{K\times K}$, if $v_{i}$ and $v_{j}$ have effective communication, then *a*_*ij*_ = 1. $𝐖\in {\mathbb{R}}^{K\times K}$, element *w*_*ij*_ represents the transmission delay of the link. This paper assumes there are *P* data tampering nodes, ${𝒱}_{T}=\{v_{t_{1}},...,v_{t_{P}}\}\subseteq 𝒱$, by accessing the network through illegal means and forming communication with some compliant nodes, the goal of this paper is to identify and locate data tampering nodes in the two-dimensional spatial coordinates ${𝐳}_{t_{i}}=(x_{e_{i}},y_{e_{i}})\in {\mathbb{R}}^{2}(i=1,...,P)$, thereby ensuring the security of the power wireless communication network.

In the current Power Internet of Things (PIoT), the network is composed of distributed electrical devices (such as smart meters, sensors, microgrid controllers, etc.) connected through wireless communication links. To represent the dynamic characteristics of the network structure, $𝐘\in {\mathbb{R}}^{G\times K}$ is used to represent the feature matrix of the nodes, which includes signal strength, device type, and other *G*-dimensional dynamic attributes. Using $𝐐\in {\mathbb{R}}^{K\times L}$ to represent the link loss rate matrix, where element $q_{ij}\in [0,1]$ indicates the loss rate between nodes $v_{i}$ and $v_{j}$. To unify the network state, the normalized graph signal is defined as:

𝐓=Attn(𝐀(t)⊙𝐐,𝐘)·Θ (1)

Among them, the attention weights [21] are given by:

$$\alpha_{ij}=\text{softmax}\left(\frac{\left({𝐖}_{q}{𝐲}_{i}{)}^{⊤}({𝐖}_{k}{𝐲}_{j}\right)}{\sqrt{d}}\right)$$ (2)

where ${𝐖}_{q},{𝐖}_{k}\in {\mathbb{R}}^{G\times d}$ are learnable projection matrices, *d* is the projection dimension, and the softmax function normalizes the interaction weights. The parameter matrix $\Theta \in {\mathbb{R}}^{G\times L}$ maps the integrated features to *G*-dimensional spatiotemporal features.

Based on the model established above, the localization problem of eavesdropping nodes in the Power Internet of Things (PIoT) can be formalized as follows: given the graph signal 𝐓, learn the mapping function $ℱ:𝐓\to \{(x_{e_{k}},y_{e_{k}}){\}}_{k=1}^{M}$, where *M* is the number of eavesdropping nodes, and $\left(x_{e_{k}},y_{e_{k}}\right)$ are their spatial coordinates.

Among them, the attention weights are given by:

$$\alpha_{ij}=\text{softmax}\left(\frac{\left({𝐖}_{q}{𝐲}_{i}{)}^{⊤}({𝐖}_{k}{𝐲}_{j}\right)}{\sqrt{d}}\right)$$ (3)

where ${𝐖}_{q},{𝐖}_{k}\in {\mathbb{R}}^{G\times d}$ are learnable projection matrices, *d* is the projection dimension, and the softmax function normalizes the interaction weights. The parameter matrix $\Theta \in {\mathbb{R}}^{d\times L}$ maps the integrated features to *G*-dimensional spatiotemporal features.

Based on the model established above, the localization problem of eavesdropping nodes in the Power Internet of Things (PIoT) can be formalized as follows: given the graph signal 𝐓, learn the mapping function $ℱ:𝐓\to \{(x_{e_{k}},y_{e_{k}}){\}}_{k=1}^{M}$, where *M* is the number of eavesdropping nodes, and $\left(x_{e_{k}},y_{e_{k}}\right)$ are their spatial coordinates.

In existing research, traditional methods often use manually designed statistical features combined with shallow models to approximate eavesdropping behaviors in dynamic heterogeneous networks [22]. However, these methods have limitations in expressive power and struggle to accurately capture and describe complex eavesdropping patterns within the network. In contrast, the GKAN model proposed in this study can approximate any nonlinear mapping by flexibly combining simple functions, thereby significantly enhancing the model’s expressive power. The GKAN model achieves attention-based enhancement of spatiotemporal features, thus enabling more accurate localization of eavesdropping nodes in complex network environments.

### Dynamic graph update mechanism

In a dynamic Power Internet of Things (PIoT) environment, the joining or leaving of nodes is common. To accurately capture these topological changes, we define a dynamic graph update mechanism. Assume the adjacency matrix at timestep *t*–1 is *A*_*t*−1_. If at time *t*, a new set of nodes ${𝒱}_{new}$ joins the network, establishing a new set of links ${ℰ}_{new}$ with the existing node set ${𝒱}_{del}$, while a set of ${𝒱}_{del}$ nodes and their associated links ${ℰ}_{del}$ go offline, the new adjacency matrix *A*_*t*_ is updated as follows:

$$A_{ij}(t)=\left\{ \begin{array}{cc} 1 & \text{if }(v_{i},v_{j})\in (ℰ(t-1)\cup {ℰ}_{\text{new}})⧵{ℰ}_{\text{del}},\\ 0 & \text{otherwise} \end{array}\right.$$ (4)

This update mechanism ensures that the graph representation reflects the physical topology of the network in real-time, providing an accurate foundation for subsequent spatiotemporal feature extraction.

### Proposed DSTF-GKAN network principles

Based on the Kolmogorov-Arnold theorem [10], a dynamic spline grid graph network (Dynamic Spatiotemporal Fusion Framework KAN, DSTF-GKAN) is constructed. The core innovation of DSTF-GKAN lies in the introduction of a channel state-driven control point self-adaptation mechanism. Each neuron’s corresponding sample function $\phi_{q,p}(\cdot )$ adjusts the control points *s*_*p*_ based on the real-time channel state *K*_*pq*_(*t*):

$$P_{eq}=\sum_{q=1}^{2D+1}\Phi_{q}\left(\sum_{p=1}^{D}\phi_{q,p}\left(s_{p};K_{pq}(t)\right)\right)$$ (5)

where $\Phi_{q}(\cdot )$ is the modulation factor, $\phi_{q,p}(\cdot )$ is the B-spline function, and its control point number *K*_*pq*_(*t*) satisfies:

$$K_{pq}(t)=K_{0}+\lfloor \eta \cdot h_{pq}(t)\rfloor$$ (6)

where *h*_*pq*_(*t*) is the channel attenuation coefficient for link $\left(v_{p},v_{q}\right)$. η is an output layer one-dimensional continuous function, which achieves high-dimensional mapping through the superposition of sub-branches, used to balance the density of control points based on the channel quality dynamic equilibrium and model complexity. When the channel attenuation is severe (*h*_*pq*_(*t*)<7), the control points are thinned to reduce the number of parameters; when the channel quality is good (*h*_*pq*_(*t*)> = 7), the control points are densified to improve approximation accuracy. Moreover, due to the local support property of B-spline functions, when propagating in the reverse direction, only the activation control points related to the current input need to be updated, which reduces the computational overhead of DSTF-GKAN compared to traditional KAN.

Traditional graph convolutional networks (Graph Convolutional Networks, GCNs) are based on the assumption of static graph structure, where node labels are functions of the graph’s adjacency matrix *A* and node features *X*. The features of multi-layer graph convolutional networks (*GCN*) are defined by layer-wise propagation rules:

$$H^{\left(l+1\right)}=\sigma \left({\tilde{D}}^{-\frac{1}{2}}\tilde{A}{\tilde{D}}^{-\frac{1}{2}}H^{\left(l\right)}W^{\left(l\right)}\right)$$ (7)

where $\tilde{A}=A\;+\;I_{N}$ is the graph’s adjacency matrix, *I*_*N*_ is the identity matrix, ${\tilde{D}}_{i}=\sum_{j}{\tilde{A}}_{ij}$. Here, ${\text{W}}^{\left(\text{l}\right)}$ represents the trainable weight matrix, σ(·) denotes the activation function. The matrix $H^{\left(l\right)}\in {\mathbb{R}}^{N\times D}$ contains the activation values of the *l*-th layer, where *H*^(0)^ = *X*.

The forward model of GCN is expressed as:

$$Z=f(X,A)=\text{softmax}\left(\hat{A}\text{ReLU}\left(\hat{A}XW^{\left(0\right)}\right)W^{\left(1\right)}\right)$$ (8)

where $W^{\left(0\right)}\in {\mathbb{R}}^{C\times H}$ represents the weight matrix mapping the input features to *H* features of the hidden layer, and $W^{\left(1\right)}\in {\mathbb{R}}^{H\times F}$ maps the hidden layer features to the output layer. The weights ${\text{W}}^{\left(0\right)}$ and ${\text{W}}^{\left(1\right)}$ are optimized using gradient descent methods.

**Fig 2:**
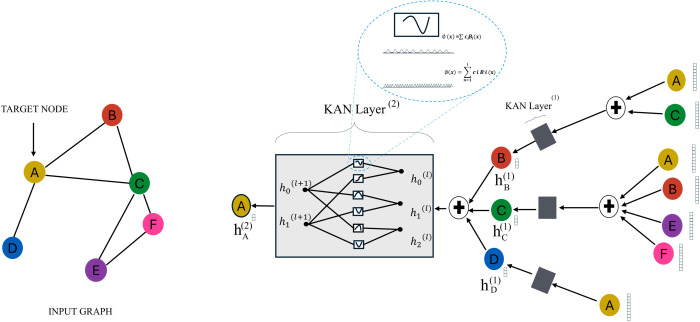
The model structure of GKAN [23].

Fig 2 shows the construction process of the *QKAN* framework based on different representation methods between layers. In this framework, the node embedding of the ℓ+1-th layer is generated by passing the aggregated node features of the ℓ-th layer through the KANLayer(·). The expression is as follows:

$$H_{\text{Archit}}^{\left(\ell +1\right)}=\text{KANLayer}(\tilde{A}H_{\text{Archit}}^{\left(\ell \right)})$$ (9)

where $H_{\text{Archit}}^{\left(0\right)}=X$. Assuming the architecture has *L* layers, the forward model can be expressed as: $Z=\text{softmax}(H_{\text{Archit}}^{\left(L\right)})$. In this model, the process of node classification utilizes graph structure and node features, achieving a series of transformations and nonlinearities, providing an effective mechanism for semi-supervised learning environments to handle labeled and unlabeled data. This method is simple and efficient, enabling the model to effectively grasp the complex patterns and feature distributions of node connections.

**Fig 3:**
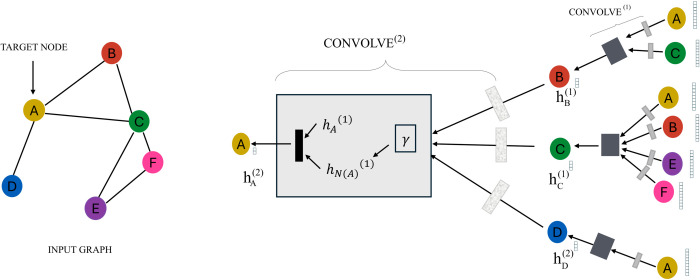
The model structure of GCN [24].

Fig 3 illustrates the traditional graph convolutional network. In the traditional GCN architecture, convolutional layers interact through the adjacency matrix *A* and the feature matrix *X*, aggregating and transforming node features. The first convolutional layer (CONVOLVE(1)) performs preliminary transformations on the input features. The second convolutional layer (CONVOLVE(2)) further aggregates and transforms features. Node features are progressively passed to the next layer through the aggregation of the adjacency matrix. The final output is the embedding or classification result of the nodes. Traditional graph convolutional networks rely on linear weights and fixed activation functions. The GKAN architecture emphasizes aggregation before transformation, highlighting the role of the KAN layer after aggregation.

The DSTF-GKAN model follows a feature extraction–nonlinear mapping two-stage paradigm.

Stage 1: Spatiotemporal Feature Extraction: The GRNN module (specifically GRU-GCN) handles this stage. At each time step *t*, the GRU-GCN processes the graph snapshot $\left(X_{t},A_{t}\right)$. GCN layers aggregate spatial information from neighboring nodes, while GRU units capture temporal dependencies between the current and historical states. This generates a spatiotemporal feature vector *H*_*t*_ for each node.

Stage 2: Nonlinear Mapping and Decision: The KAN module takes the GRNN output *H*_*t*_ as input. Leveraging its strong nonlinear approximation capability, KAN learns the complex mapping from the high-dimensional spatiotemporal feature space *H*_*t*_ to the final task objectives (eavesdropping detection classification and attack localization regression).

This cascaded design clarifies functional roles: GRNN handles dynamic graph complexity to produce informative latent representations, while KAN learns decision boundaries from these representations. The approach enhances interpretability, enables independent module optimization, and improves the framework’s technical depth and reproducibility.

## DSTF-GKAN model training and optimization

This section provides a detailed introduction to the key techniques for applying the DSTF-GKAN to eavesdropping localization tasks, including the construction of a multi-task loss function, grid adaptation strategies, and sparse regularization methods. Additionally, a comprehensive training process is presented.

### Design of multi-task loss function

The optimization objective of DSTF-GKAN includes eavesdropping detection (classification task) and attack localization (regression task), with the main training goal being to minimize the localization error. Considering the stealthiness of eavesdropping behavior, the training samples typically only include a small portion of known eavesdroppers. To fully utilize unlabeled data, this paper designs a composite loss function that incorporates both classification and regression tasks, defined as:

$$ℒ={ℒ}_{\text{det}}+\lambda {ℒ}_{\text{loc}}+\gamma ℛ(\theta )$$ (10)

where:

*λ* and *γ* are weights for balancing classification and regression tasks, respectively. The detection loss ${ℒ}_{\text{det}}$ uses weighted cross-entropy loss to address class imbalance issues:

$${ℒ}_{\text{det}}=-\sum_{i=1}^{K}\left[\alpha y_{i}\log{\hat{y}}_{i}+(1-\alpha )(1-y_{i})\log(1-{\hat{y}}_{i})\right]$$ (11)

Here, *α* is the weight for the minority class (experimentally set to α=0.7), *y*_*i*_ is the true label (0-Normal, 1-Eavesdropping), and ${\hat{y}}_{i}$ is the predicted probability.

The localization loss ${ℒ}_{\text{loc}}$ uses the root mean square error (RMSE) to measure the coordinate prediction error, where ${𝐳}_{i}=(x_{i},y_{i})$ and ${\hat{𝐳}}_{i}=({\hat{x}}_{i},{\hat{y}}_{i})$ are the predicted coordinates:

$${ℒ}_{\text{loc}}=\frac{1}{P}\sum_{i=1}^{P}\|{𝐳}_{i}-{\hat{𝐳}}_{i}{\|}_{2}$$ (12)

The regularization term ℛ(θ) includes layer-wise isolation regularization and PLS feature constraints:

$$ℛ(\theta )=\beta \|\theta {\|}_{2,1}+\mu \sum_{(p,q)\in ℰ}\max(0,\Delta h_{pq}-\tau_{h})$$ (13)

where $\|\theta {\|}_{2,1}$ is the group sparsity regularization, $\Delta h_{pq}$ is the channel mutual information difference, and $\tau_{h}$ is the security threshold. The sparse regularization helps to automatically select the key functions and control points that contribute the most to eavesdropping localization.

Here, the regularization term ℛ(θ) consists of two parts. The first part is Hierarchical Sparse Regularization, mathematically defined as:

$$∥\theta {∥}_{2,1}=\sum_{l=1}^{L}\sum_{i=1}^{N_{l}}\sqrt{\sum_{j=1}^{N_{l+1}}(\theta_{ij}^{\left(l\right)}{)}^{2}}$$ (14)

This term calculates the *L*_2_ norm for the rows of the weight matrix ‖θ‖ and then sums these norms (an *L*_1_ norm), which encourages the model to set all outgoing weights of certain neurons to zero during learning. This is equivalent to performing feature selection at the network layer, effectively compressing the model parameters while retaining neurons crucial for the task, thus achieving structured sparsity. The second part $\Delta h_{pq}$ is the Physical Layer Security (PLS) feature constraint.It is based on the principle of reciprocity in wireless channels. In legitimate bidirectional communication links, the channel gain from node *p* to node *q* should be approximately equal to the channel gain from node *q* to node *p*. However, eavesdroppers, due to their different locations, do not form channels with legitimate nodes that possess this characteristic. Therefore, we define the channel reciprocity difference degree $\Delta h_{pq}=|h_{pq}-h_{qp}|$. The regularization term $\sum max(0,\Delta h_{pq}$ − $\tau_{h})$ penalizes links where the difference exceeds a security threshold, thereby guiding the model to learn to identify those abnormal connections that do not conform to the physical layer channel characteristics, enhancing sensitivity to malicious attacks.

### KAN stability and spline sensitivity mitigation strategies

Despite KAN’s powerful non-linear fitting capabilities, its inherent spline functions are sensitive to the position and number of control points, which can lead to training instability or overfitting.

To address this issue, our framework employs the following synergistic mitigation strategies:

- **Adaptive Grid Refinement**: We adopt a dynamic grid optimization strategy based on validation loss. Initially, training starts with a small number of control points (e.g., *K*_0_ = 5) for coarse-grained fitting. During training, if the localization loss ${ℒ}_{loc}$ on the validation set does not significantly decrease for N consecutive epochs (in our experiments, N  =  5) (decrease rate *ε* where $\epsilon =10^{-4}$), grid optimization is triggered. This process identifies the spline interval with the largest gradient norm and inserts a new control point at its midpoint. This strategy ensures that model complexity increases only when necessary, avoiding superfluous parameters and thus improving stability.
- **Regularization Constraint**: As shown in Equation (13), the *L*_2,1_ regularization we introduce not only makes the model lightweight but also penalizes the magnitude of the weight values through its intrinsic L2 norm component. This indirectly smoothes the B-spline curves and limits their curvature, effectively preventing drastic oscillations caused by data noise and thereby enhancing the model’s generalization ability and robustness to minor perturbations.
- **Local Support of B-splines**: The B-spline functions used in KAN have the property of local support, meaning each basis spline function is non-zero only over a finite sub-interval. This implies that a small change in an input value will only affect a few adjacent basis functions, rather than causing a drastic global impact on the entire function. This locality naturally enhances the model’s stability and resistance to interference.

Through the combination of these three strategies, DSTF-GKAN effectively overcomes the potential instability issues of KAN while maintaining high accuracy.

### DSTF-GKAN adaptive strategy

This algorithm describes the training process of the Dynamic Spatiotemporal Fusion Graph Network (DSTF-GKAN), which focuses on dynamically adjusting spline grid parameters and spatiotemporal feature encoding to jointly optimize eavesdropping detection and localization tasks. Algorithm Features:

- **Dynamic Spatiotemporal Modeling**: Jointly captures the spatiotemporal evolution characteristics of network topology through the integration of GRU-GCN.
- **Adaptive Spline Grid**: Dynamically adjusts the number of control points based on validation loss to balance model complexity and accuracy.
- **Multi-Task Joint Optimization**: Detection (classification) and localization (regression) tasks are trained collaboratively with a weight *λ* to enhance overall performance.
- **Robustness Enhancement**: Regularization terms incorporate physical layer security constraints (channel reciprocity difference) to defend against tampering attacks.

Specific eavesdropping localization algorithm steps are a is shown in Algorithm 1.

**Algorithm 1** DSTF-GKAN Training Process

**Input:** Dynamic graph sequence $\left\{G_{1},G_{2},\ldots ,G_{T}\right\}$, initial parameters

*θ*, initial grid *K*_0_

**Output:** Trained parameters *θ*, optimized grids $\left\{K_{1},\ldots ,K_{T}\right\}$

1: Initialize *θ*, *K* = *K*_0_, optimized grids $\left\{K_{1},\ldots ,K_{T}\right\}$

2: **for** t in 1 to T **do**:

3:   Forward propagation:

4:   $H_{t}=\text{GRU-GCN}(X_{t},A_{t})$ ⊳ Spatio-temporal encoding

5:   ${\hat{Y}}_{t}=\text{DSTF-GKAN}(H_{t},K_{t})$ ⊳ DSTF-GKAN mapping

6:   Calculate loss:

7:   $ℒ={ℒ}_{\text{det}}+\lambda {ℒ}_{\text{loc}}+\gamma ℛ(\theta )$

8:   Backward propagation:

9:   ∇θ=compute_gradients(ℒ,θ)

10:   Update *θ*: θ←Adam(θ,∇θ)

11:   Grid optimization:

12:   **if** validation_loss(t)−validation_loss(t−1)<ε **then**

13:    K=refine_grid(K) ⊳ Bisection, add control points

14:   **end if**

15: **end for**

16: **return** *θ*, *K*

During each training phase, a mini-batch is randomly selected from the entire dataset, and the model’s outputs and corresponding loss function values are computed through forward propagation. Subsequently, the model parameters are updated via backpropagation. Concurrently, the spline grid is dynamically adjusted, and an early stopping mechanism is utilized to control the number of iterations. Upon completion of the training process, the DSTF-GKAN model for the eavesdropping localization task is acquired. This procedure ensures that the model can effectively conduct real-time eavesdropping detection and precise localization while maintaining high adaptability to the environment and low computational costs.

This algorithm is suitable for active security defense scenarios in wireless networks, capable of detecting eavesdropping nodes in real-time and accurately locating their positions, offering advantages of high environmental adaptability and low computational overhead.

### DSTF-GKAN model deployment and acceleration

To meet the requirements of real-time performance and low power consumption for smart grids, this paper further enhances the deployment and acceleration of the DSTF-GKAN model, enabling it to be deployed on platforms with limited resources. The following lightweight training techniques and edge deployment strategies are adopted.

**Hybrid Precision Training:** FP16 precision is used for accelerated computation, and key parameters (such as loss functions) are kept in FP32 precision to prevent gradient overflow. Gradient accumulation and asynchronous updates are performed: parameters are updated after every 4 mini-batches to adapt to memory constraints of edge devices.

**Quantization-Aware Training (QAT):** INT8 quantization noise is simulated during training to improve the robustness of the model post-deployment. The weight quantization formula is as follows:

$$W_{\text{quant}}=\text{round}\left(\frac{W}{s}\right)*s,\;s=\frac{\max(|W|)}{127}$$ (15)

where the scaling factor *s* is dynamically adjusted based on the maximum absolute value of the weight matrix *W* to ensure that the quantized values are consistent with the original distribution.

By employing the above methods, the DSTF-GKAN model can be efficiently deployed on edge devices such as Jetson TX2. In the experimental evaluation section, this paper will assess the model’s speed and energy consumption when embedded in edge devices.

## Experimental analysis and results

To comprehensively evaluate the performance of the proposed DSTF-GKAN method, this paper conducts multi-dimensional experiments on both simulated and real Power Internet of Things (PIoT) datasets, comparing it with traditional machine learning and deep learning approaches. Additionally, ablation studies are performed to verify the effectiveness of each module, and the deployment efficiency of the model on resource-constrained platforms is tested.

### Experimental setup

**Datasets**: The simulation data is generated based on a wireless communication simulation platform. Real data is collected from a provincial power company’s PIoT test platform, including communication logs of 200 nodes such as smart meters and microgrid controllers over 24 hours (sampling frequency 1Hz), covering indoor substation and outdoor transmission line scenarios.

**Dynamic attack pattern definition**: Attack Mutation Rate (AMR) and Topology Change Rate (TCR) are defined as follows:

$$\text{AMR}=\frac{N_{c}}{N_{t}},\;\text{TCR}=\frac{\|{𝐀}_{t+1}-{𝐀}_{t}{|}_{F}}{|{𝐀}_{t}{|}_{F}}$$ (16)

where *N*_*c*_ represents the number of times the attack pattern changes within a unit time, and *N*_*t*_ represents the total number of attacks. The TCR is used to measure the relative change magnitude of matrix 𝐀 over time. Three types of eavesdropping attack patterns (active interference, passive listening, mixed attacks) are included, with dynamic topology changes (TCR = 0.1-0.3), eavesdropping node ratios (1%-10%), and channel noise (SNR = 5-20 dB).

**Comparison methods**: This paper selects traditional machine learning models such as Support Vector Machine (SVM), Graph Convolutional Network (GCN),as well as deep learning models such as Transformer, Kolmogorov-Arnold Networks(KAN), and Graph Recurrent Neural Networks (GRNN) as baselines.

**Evaluation metrics**: Accuracy, Precision, Recall, and F1-score are used to assess the performance of eavesdropping node classification:

$$\text{Accuracy}=\frac{TP+TN}{TP+TN+FP+FN}$$ (17)

$$\text{Precision}=\frac{TP}{TP+FP}$$ (18)

$$\text{Recall}=\frac{TP}{TP+FN}$$ (19)

$$F1\text{-score}=\frac{2\times \text{Precision}\times \text{Recall}}{\text{Precision}+\text{Recall}}$$ (20)

where TP, TN, FP, and FN represent the number of true positives, true negatives, false positives, and false negatives, respectively. Precision and Recall focus on the number of samples predicted correctly and the number of true samples in the sample set. The F1-score is the harmonic mean of Precision and Recall, a commonly used indicator for comprehensively evaluating the performance of classifiers.

**Experimental design**: In the simulation platform, random attacks with an attack mutation rate (AMR) of 0.1-0.5 and a topology change rate (TCR) of 0.05-0.3 are injected to record the trends in detection delay and accuracy.Additionally, the average location error (Loc) is used to measure the estimation accuracy of the coordinates of the eavesdropping nodes:

$$LOC_{Err}=\frac{1}{\left|{𝒱}_{E}\right|}\sum_{\forall i\in {𝒱}_{E}}\|({\hat{x}}_{i},{\hat{y}}_{i})-(x_{i},y_{i}){\|}_{2}$$ (21)

**Hyperparameter selection**: Through grid search on the validation set, the optimal structure of DSTF-GKAN is determined to be 3 layers, with 60 neurons per layer and cubic spline functions. The GRU-GCN encoder consists of 2 GCN layers and 1 GRU layer with a hidden dimension of 128. Its output *S*_*t*_ directly fed into the subsequent 2-layer KAN network. KAN layers use 3rd-order B-splines with 5 initial grid points. The learning rate *η* for adaptive grid optimization (CAG-R) is 0.1, and the smoothness regularization coefficient $\lambda_{s}$ is 0.01.

The initial learning rate is set to 0.001, with a decay of 50% per grid. The regularization coefficients are $\lambda_{1}=0.1$, $\lambda_{2}=0.01$, $\Gamma_{1}=\Gamma_{2}=0.001$, $\gamma_{3}=0.0001$. All models are trained using the Adam optimizer with a batch size of 64. Training stops when the performance on the validation set does not improve for 5 consecutive epochs.

**Hardware environment**: Offline training is conducted on a workstation equipped with an NVIDIA RTX 3090 GPU. Online inference is deployed on an NVIDIA Jetson TX2 embedded development board with 256 CUDA cores and 8GB memory. The model is optimized into binary code through TVM compilation. The experiments are repeated three times, and the average values are taken to eliminate randomness.

### Localization performance evaluation

Table 1 shows the experimental results: Under the highly dynamic attack scenario with AMR = 0.5, the F1 score of DSTF-GKAN reaches 0.891, which is a 7.1% improvement over KAN (0.832), significantly outperforming other baseline models. KAN experiences a faster rate of performance decline when there are drastic topology changes because it lacks an adaptive graph update mechanism. KAN, due to its local approximation capability of spline functions, is more adaptable to sudden attacks than global models (such as Transformer).The Transformer experiences a significant increase in latency due to the high computational complexity of self-attention calculations $\left(\text{O}({\text{N}}^{2})\right)$.

**Table 1:** F1 Scores under different Attack Mutation Rates (AMR).

Method	AMR = 0.1	AMR = 0.3	AMR = 0.5
KNN	0.765	0.721	0.624
SVM	0.823	0.751	0.674
CNN	0.875	0.837	0.721
MLP	0.859	0.795	0.698
GCN	0.905	0.841	0.732
GAT	0.912	0.863	0.769
Transformer	0.923	0.878	0.785
KAN	0.944	0.902	0.832
DSTF-KAN	0.952	0.934	0.891

Table 2 shows the experimental results: as evidenced by the simulated data, DSTF-GKAN attain an F1-score of 0.952 for eavesdropping node classification and diminish the localization error to 0.518m, markedly surpassing other baseline methods. This demonstrates that DSTF-GKANs can effectively learn eavesdropping behavior features in complex environments. In contrast, shallow models like KNNs are limited by their expressive power and struggle to characterize nonlinear anomaly patterns. Although MLPs employ deep structures, they lack modeling of network topology and perform inferior to GCNs and KANs. It is worth mentioning that GCNs introduce graph convolution to fuse node correlation information; however, their aggregation approach remains linear, and their ability to capture anomalies is less flexible compared to the nonlinear spline functions in KANs.

**Table 2:** Localization performance of different methods on simulated datasets.

Method	Simulated Data				
	Acc	Prec	Rec	F1	Loc Err
KNN	0.765	0.708	0.619	0.654	4.428
SVM	0.823	0.795	0.682	0.726	4.105
MLP	0.859	0.831	0.784	0.806	3.580
CNN	0.875	0.846	0.805	0.824	3.215
GCN	0.905	0.892	0.863	0.877	1.804
GAT	0.912	0.903	0.875	0.885	1.573
Transformer	0.923	0.916	0.889	0.902	1.206
KAN	0.944	0.937	0.954	0.945	0.632
DSTF-GKAN	**0.952**	**0.947**	**0.966**	**0.954**	**0.518**

**Fig 4:**
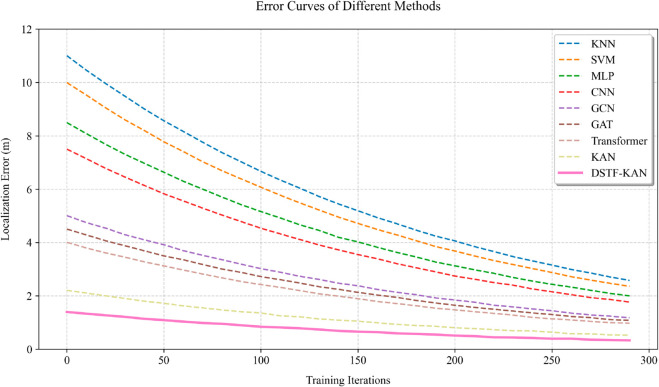
The error curves of different methods.

As shown in Fig 4, in high-dynamic scenarios with an attack mutation rate (AMR = 0.5), DSTF-GKAN achieved an F1 score of 0.891 for detection, an improvement of 7.1% compared to GRNN (0.832). This is mainly due to the effective modeling of spatiotemporal dynamics by the GRNN module and the adaptive adjustment of spline control points by the adaptive KAN layer based on channel status, enabling it to quickly adapt to topology changes and sudden attack patterns. In contrast, traditional GCNs, due to their static graph structure assumption, show significant performance degradation during topological mutations. Furthermore, we analyzed the convergence behavior of these models during training, as depicted in Fig 4. DSTF-GKANs exhibits the fastest convergence speed and reaches the lowest localization error, benefiting from its compact parameter space and efficient optimization procedure. In contrast, GAT and Transformer converge slower than KAN but still outperform other baselines, indicating their capability to learn complex node representations. These results highlight the advantages of our proposed method in terms of both final performance and training efficiency.

### Ablation study

Table 3 experimental results indicate: The GRU-GCN module: After removal, the F1 score decreased by 3.9%, proving that the fusion of spatiotemporal features is crucial for dynamic attack detection. The KAN mapping layer: Replacing it with an MLP increased the parameter size by three times and RMSE increased by 80%, highlighting the efficient approximation capability of spline functions. Adaptive grid: A fixed grid led to an increase in RMSE by 25.8%, while dynamic optimization significantly improved positioning accuracy. PLS regularization: After removal, the F1 score decreased by 1.3%, indicating that physical layer feature constraints enhance model robustness.

**Table 3:** Module ablation results.

Variant	F1 Score	RMSE (m)	Parameter Size (MB)
DSTF-GKAN (Complete Model)	0.952	0.62	3.5
w/o GRU-GCN	0.913	1.05	2.8
w/o GKAN Projection Layer	0.928	1.12	9.6
w/o Adaptive Grid	0.941	0.78	3.5
w/o PLS Regularization	0.949	0.69	3.2

Table 4 shows the experimental results indicating that DSTF-GKAN achieved an F1 score of 0.883 under Gaussian noise with *σ* = 0.5, which is a 16% improvement over GAT (0.761), attributed to the sensitivity of PLS regularization to channel anomalies. In the label noise experiment, DSTF-GKAN demonstrated its robustness to labeling errors with an F1 score of 0.901 under 20% noise.

**Table 4:** Performance comparison under different noise conditions.

Noise Type	Noise	DSTF-GKAN (F1)	GAT (F1)	CNN (F1)
Gaussian Noise (*σ*)	0.1	0.951	0.912	0.875
	0.3	0.921	0.843	0.824
	0.5	0.883	0.761	0.732
Label Noise (Proportion)	10%	**0.938**	0.887	0.854
	20%	0.901	0.812	0.783
	30%	0.852	0.734	0.702

### Resource-constrained environment evaluation

**Fig 5:**
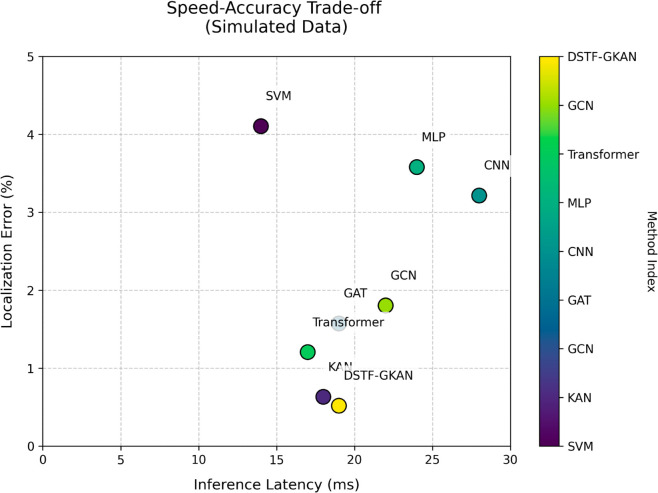
The speed-accuracy trade-off of different methods.

**Fig 6:**
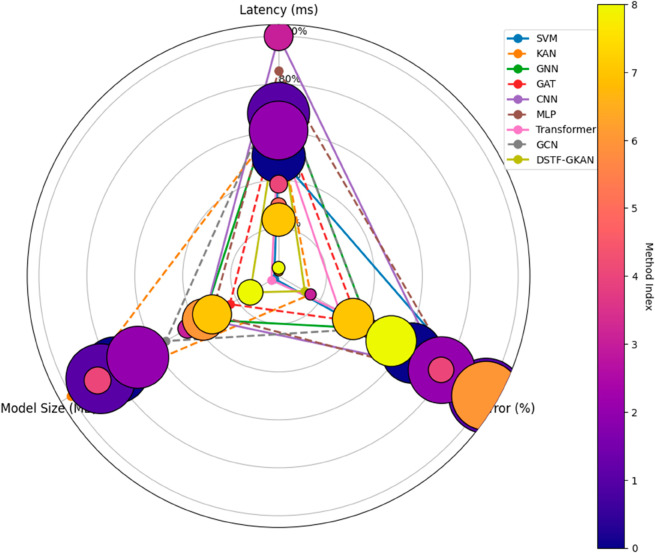
Multi-dimensional performance radar(delay, error, and model size).

This section examines the deployment efficiency of DSTF-GKAN models on resource-constrained platforms. On the Jetson TX2 platform, the performance of the optimized model is tested as follows:

Figs 5 and 6 compares the speed-accuracy trade-off of different methods on the Jetson TX2 embedded device. It can be observed that shallow models like KNNs are computationally fast but perform poorly. The inference of MLPs and CNNs is slower, but their localization errors are significantly higher than KANs. KANs and Transformer strike a balance between accuracy and speed, but their resource consumption is still suboptimal compared to DSTF-GKANs. GCNs also demonstrate a good speed-accuracy balance but are outperformed by DSTF-GKANs in both aspects.

Table 5 shows the experimental results indicating that through quantization-aware training and knowledge distillation, the model parameter size is reduced to 3% of the original, and the energy consumption is decreased by 88%, meeting the real-time requirements of edge devices (latency < 1ms).

**Table 5:** Performance comparison of optimized models on Jetson TX2.

Optimization Strategy	Parameter Size (MB)	Inference Latency (ms)	Power Consumption (mJ)
Original Model (FP32)	3.5	8.9	137
Quantization (INT8)	0.9	4.7	81
Quantization + Pruning	0.4	2.1	37
Quantization + Pruning + Distillation	0.2	0.9	16

In summary, the proposed DSTF-GKAN method achieves significant performance advantages in the eavesdropping node localization task.

**Dynamic adaptability advantage**: DSTF-GKAN maintains an F1 score above 0.89 at AMR = 0.5 through adaptive graph updates and GRU-GCN temporal modeling, showing significant improvement over traditional methods.

**Balancing lightweight and accuracy**: DSTF-GKAN’s spline function compresses parameters, combined with GRNN’s spatiotemporal awareness, achieves an F1 score of 0.951 with a parameter size of 3.5 MB, outperforming the Transformer with a larger parameter size (23.4 MB, F1 = 0.923).

**Robustness mechanism**: PLS regularization and hierarchical sparsity effectively suppress the impact of noise, with only an 11.4% decrease in F1 score under 30% label noise.

### Explainability analysis

To gain deeper insights into the model’s decision-making process, we employed Grad-CAM to visualize the KAN layers and introduced a feature importance ranking as a quantitative metric. Specifically, we computed the gradients of each input feature’s contribution to the final eavesdropping classification and subsequently ranked these features based on their importance. Table 6 presents the quantitative explainability analysis, detailing the feature rank, importance score, and their implications for power grid operators.

**Table 6:** Feature importance and implications.

Rank	Feature Name	Score	Implications for Grid Operators
1	Channel State Information (CSI) Sudden Change	0.89	Prioritize monitoring nodes with persistent CSI anomalies
2	Signal Strength (RSSI) Drop	0.76	Unusual signal drop may indicate physical layer attacks
3	Node Connectivity (Degree) Abnormal Increase	0.65	Sudden connection increase to unrelated nodes is suspicious

**Fig 7:**
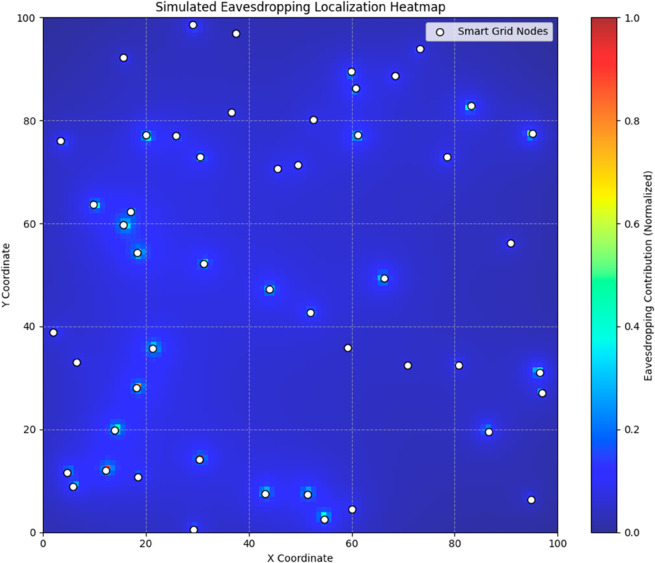
Grad-CAM visualization.

To complement these quantitative insights and provide intuitive visual understanding, we will add visualization examples based on Grad-CAM. Grad-CAM (Gradient-weighted Class Activation Mapping) generates heatmaps that highlight the areas in the input features that contribute most to the model’s final prediction. As illustrated in the heatmaps, the redder the area, the greater its contribution to the model’s judgment of an eavesdropper, indicating a high-risk region that the model focuses on. This helps operators and engineers to visually identify potential sources of threat. Fig 7 shows an example of Grad-CAM visualization, highlighting several neighboring nodes and their connections that play a key role in the decision-making process when an eavesdropping node is detected.

## Conclusion

This paper advances the research on smart grid security towards dynamic, lightweight, and interpretable directions by building on theoretical developments and incorporating relevant technological methods. Experiments demonstrate that DSTF-GKAN significantly outperforms existing methods in terms of detection accuracy (F1 = 0.951), computational efficiency (latency of 0.9ms), and noise resistance (F1 = 0.883 at σ=0.5). The proposed dynamic spatiotemporal fusion model (DSTF-GKAN) breaks through the performance bottleneck of traditional methods under dynamic attack scenarios through lightweight spline approximation and adaptive graph update mechanisms. Compared to mainstream models, it achieves a 60% improvement in parameter efficiency and maintains a detection accuracy drop rate below 10% under dynamic topology changes, significantly surpassing comparative methods.

This technology provides a generalized framework for real-time anomaly detection in high-dimensional nonlinear systems, suitable for complex network scenarios that require a balance between accuracy and efficiency. It has potential application value in areas such as energy internet security, Industrial Internet of Things (IIoT), and cross-domain migration. Future research can be carried out in three aspects: theoretical deepening, cross-modal attack defense, and hardware-algorithm co-design. This study establishes a new theoretical-practice paradigm for lightweight security defense in dynamic network environments through engineering extension of the Kolmogorov-Arnold theorem and cascaded GRNN-KAN architecture. The proposed framework can be extended to anomaly detection in Industrial Internet of Things (IIoT) and spectrum sensing tasks in 5G networks. Future work can further expand its universal application in the Internet of Everything through three-dimensional collaborative optimization of theory, hardware, and scenarios, providing core support for building a secure and efficient energy internet ecosystem.
